# First record on the biology of *Sarcophaga* (Bulbostyla) (Diptera, Sarcophagidae)

**DOI:** 10.3897/zookeys.909.46488

**Published:** 2020-02-05

**Authors:** Pierre-Marc Brousseau, Marjolaine Giroux, I. Tanya Handa

**Affiliations:** 1 Département des sciences biologiques, Université du Québec à Montréal, 141, avenue du Président-Kennedy, Montréal, QC, H2X 1Y4, Canada Université du Québec à Montréal Montréal Canada; 2 Montréal Insectarium / Space for life, 4581, rue Sherbrooke Est, Montréal, QC, H1X 2B2, Canada Montréal Insectarium / Space for life Montréal Canada

**Keywords:** feeding behaviour, flies, host, millipedes, Nearctic Region, Spirobolidae

## Abstract

A first breeding record for Sarcophaga (Bulbostyla) cadyi Giroux & Wheeler on the American giant millipede *Narceus americanus* (de Beauvois) (Spirobolida, Spirobolidae) is reported. Digital photographs of the terminalia of S. (B.) cadyi and of Sarcophaga (Bulbostyla) yorkii Parker are also provided.

## Introduction

*Sarcophaga* Meigen is a large and diverse genus comprising about 890 valid species worldwide (Buenaventura et al. 2017). In Canada, 39 species are currently known including 21 species recorded in Quebec (Pape 1996; Dahlem and Naczi 2006; Giroux and Wheeler 2010). Adult flies of *Sarcophaga* feed on various resources including sugar, carrion and dung. The main resources of the larvae are dead arthropods, snails and small vertebrates which they can use as scavengers, parasitoids or predators (Pape 1996; Coupland and Barker 2004; Mello-Patiu 2016). Within this genus, the recently described subgenus Bulbostyla Giroux & Wheeler comprises nine species restricted to North and South America. It differs from other *Sarcophaga* mainly by characters of the male genitalia (Giroux and Wheeler 2010). The ecology of *Bulbostyla* species remains little known, although most specimens were collected on hilltops (Giroux and Wheeler 2010). No feeding records have previously been documented for larvae.

Here, we present the first observation of an interaction between the flesh fly S. (Bulbostyla) cadyi Giroux & Wheeler and the American giant millipede *Narceus americanus* (de Beauvois) (Spirobolida, Spirobolidae). We also present digital photographs of the male terminalia of both species of *Bulbostyla* found in the province of Quebec (S. (B.) cadyi and S. (B.) yorkii Parker) and photographs of the female external terminalia of S. (B.) cadyi.

## Materials and methods

A dead *N. americanus* colonized by eight sarcophagid larvae was collected on August 20, 2017. The millipede was found on the forest floor (45°33.18'N, 73°18.30'W) at Mont-Saint-Bruno National Park in southern Quebec. The millipede and the larvae of *S. cadyi* were brought to the laboratory and kept at constant room temperature (~20 °C) in a small plastic container with garden soil. Once adult flies emerged, they were killed in the freezer and preserved in 70% alcohol. In order to be morphologically identified, they were rinsed twice in 100% ethyl acetate, then dried and pinned.

The specimens of S. (B.) yorkii were collected using a hand-held entomological net at the summit of Mont Rigaud (45°27.96'N, 74°19.56'W, summers of 2007 and 2017) and of Mont-Saint-Bruno (45°33.12'N, 73°19.68'W, summer 2010). Those specimens were killed using ethyl acetate fumes and pinned shortly afterwards.

**Figure 1.**
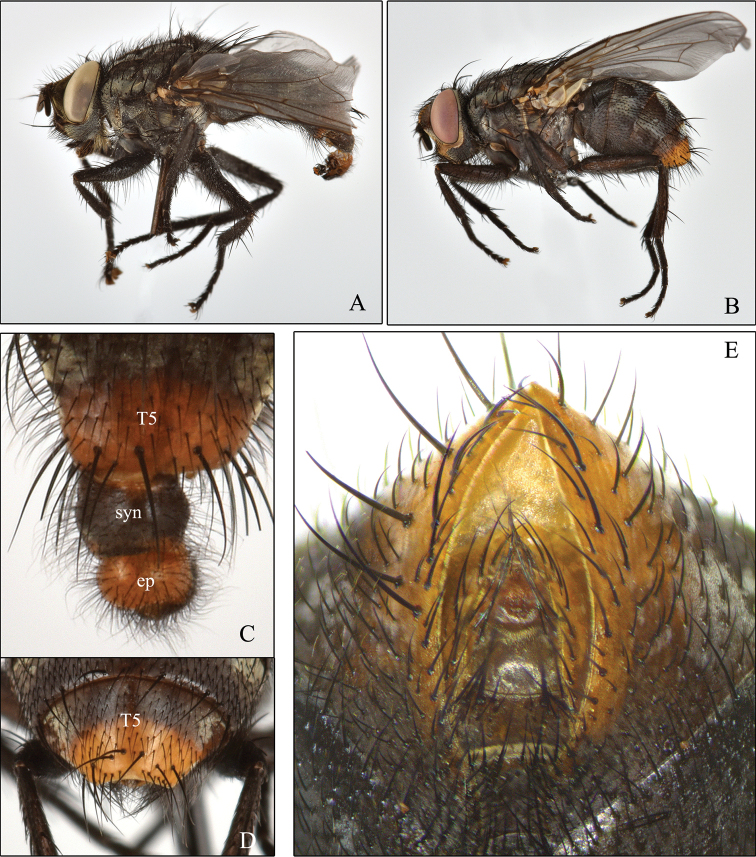
Sarcophaga (Bulbostyla) cadyi**A** male habitus **B** female habitus **C** male tergite 5 (T5), syntergosternite 7+8 (syn) and epandrium (ep), dorsal **D** female tergite 5 (T5), dorsal **E** female postabdomen, ventral.

**Figure 2.**
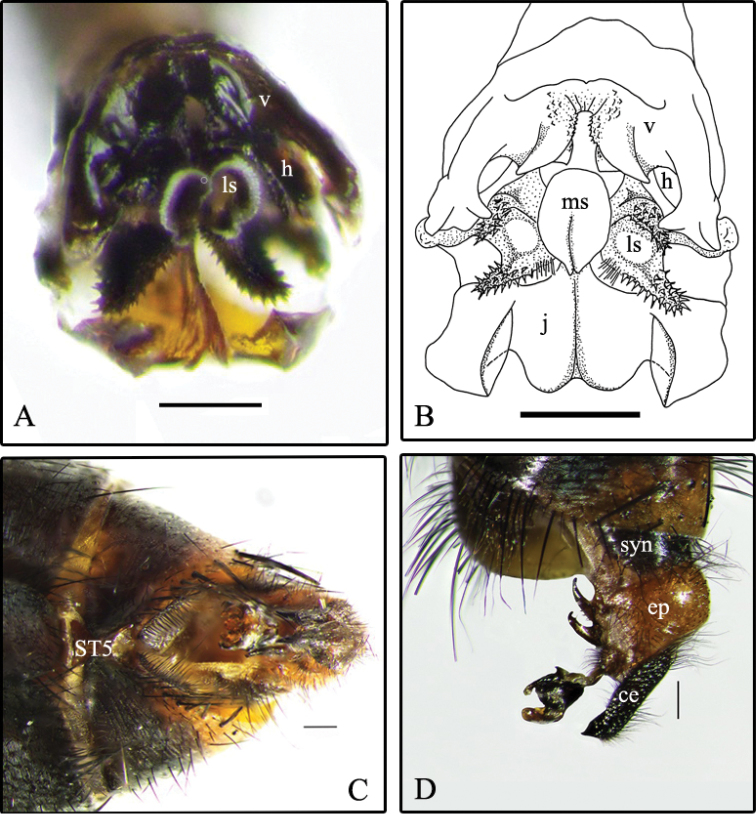
Sarcophaga (Bulbostyla) cadyi**A** distiphallus, anterior **B** distiphallus, anterior (from Giroux and Wheeler 2010) **C** male postabdomen, ventral **D** male postabdomen, left lateral. Abbreviations: j, juxta; ls, lateral stylus; ms, median stylus; h, harpes; v, vesica; syn, syntergosternite 7+8; ep, epandrium; ce, cercus; ST5, sternite 5. Scale bars: 0.2 mm (**A–B**), 0.5 mm (**C–D**).

**Figure 3.**
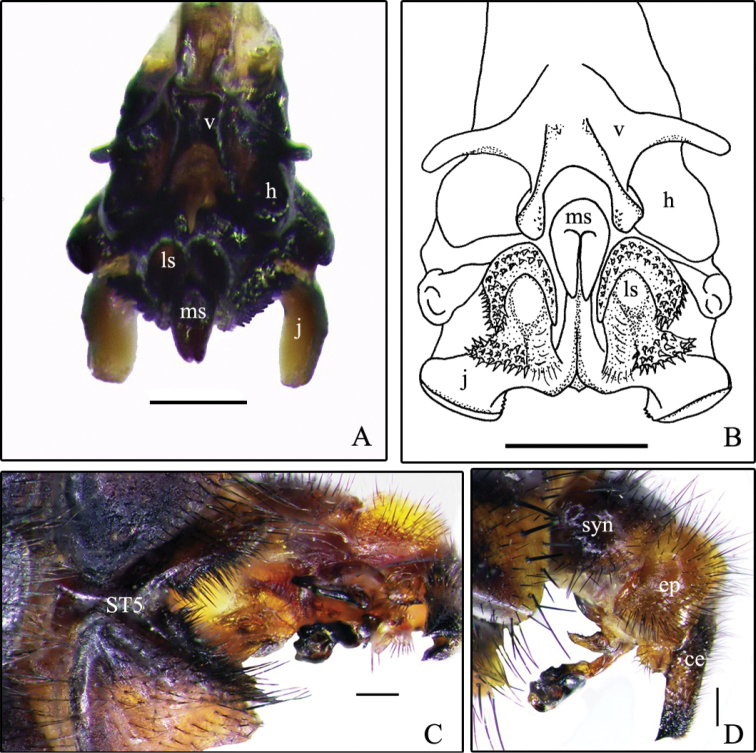
Sarcophaga (Bulbostyla) yorkii**A** distiphallus, anterior **B** distiphallus, anterior (from Giroux and Wheeler 2010) **C** male postabdomen, ventral **D** male postabdomen, left lateral. Abbreviations: j, juxta; ls, lateral stylus; ms, median stylus; h, harpes; v, vesica; syn, syntergosternite 7+8; ep, epandrium; ce, cercus; ST5, sternite 5. Scale bars: 0.2 mm (**A–B**), 0.5 mm (**C–D**).

**Figure 4.**
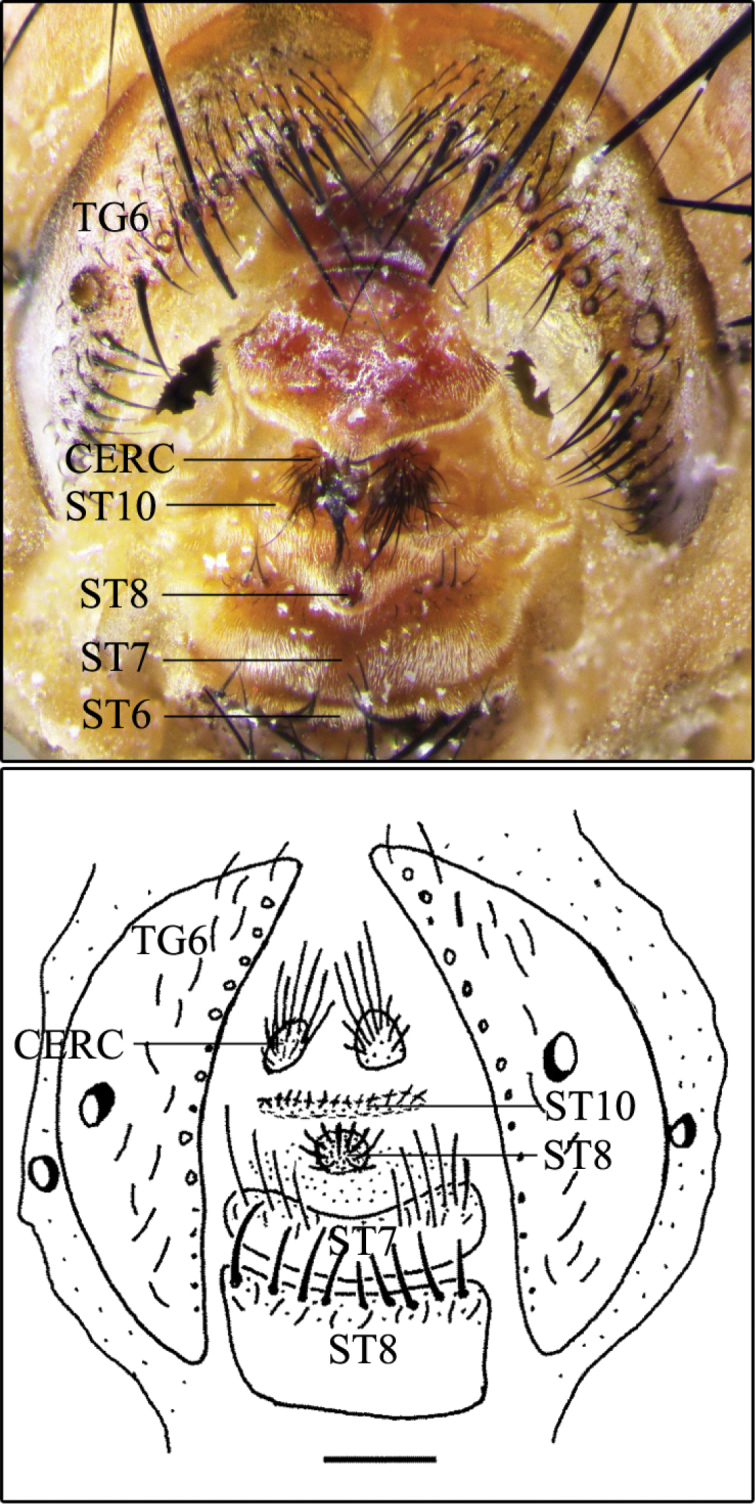
Sarcophaga (Bulbostyla) cadyi**A** female external terminalia, dorsoventral **B** female external terminalia, dorsoventral (from Giroux and Wheeler 2010). Abbreviations: cerc, cerci; st, sternite; tg, tergite. Scale bars: 0.5 mm (**A–B**).

The habitus photographs (Figs 1–4) were taken using a Nikon D810 DSLR camera with Nikon Micro-Nikkor 200 mm f/4 lens on a Manfrotto 454 micrometric positioning sliding plate. Lighting was provided by two Nikon SB-25 flash units with a Cameron Digital diffusion photo box. Adobe Photoshop Elements 13 was used as post-processing software. Photographs of the terminalia and genitalia were taken with an Olympus DP27 camera mounted with stereoscope SZX16. Images were captured and stacked using Helicon Focus 7 before being enhanced using Adobe Photoshop CC (version 20.0) (Adobe Systems, Mountain View, CA).

To solidify species identity, a leg of some specimens of S. (B.) cadyi and S. (B.) yorkii were submitted to LifeScanner (http://lifescanner.net/) and others to the Canadian Centre for DNA Barcoding for DNA barcoding. It was only possible to obtain sequences for S. (B.) cadyi. Those sequences were compared and analysed using the Barcode of Life Data (BOLD: http://boldsystems.org/) System ID Engine (Ratnasingham and Hebert 2007). Individual sequences from the successful specimens are publicly available via GenBank accession codes MK585627–MK585630. They can also be retrieved from BOLD in the public dataset DS-SARCOPH (https://doi.org/10.5883/DS-SARCOPH).

The terminology of the terminalia follows Buenaventura and Pape (2017). All voucher specimens are deposited in Insectarium de Montréal’s scientific collection (IMQC).

## Results and discussion

We present the first breeding record for a species of *Bulbostyla* and the first mention of their larvae developing in a spirobolid millipede. We also present the first mention of a *Sarcophaga* species showing a feeding interaction with a millipede in North America, and the second worldwide after the European species Sarcophaga (Myorhina) iulicida Pape (Pape 1990a).

Only three dipteran families (Sarcophagidae, Phoridae, Sciomyzidae) have been reported as parasitoids of diplopods (Hash et al. 2017). Within the Sarcophagidae, the species *Blaesoxipha beameri* Hall (Pape 1994) and species of the genus *Spirobolomyia* Townsend have been bred exclusively from spirobolid millipedes (Aldrich 1916; Pape 1990b, 1996) in North America. However, it is likely that species of this genus are not true millipede parasitoids. All observations of larviposition by *Spirobolomyia* species were on injured hosts with wounds large enough for the larva to enter (Hash et al. 2017).

We did not observe the larviposition of S. (B.) cadyi on *N. americanus*, which was already dead and colonized by the last instar larvae when we found it. Thus, we do not know if the spirobolid millipede was healthy, injured or already dead upon arrival of the female sarcophagid fly. In this sense, further investigations are needed to be able to determine the larval feeding habits of S. (B.) cadyi. The larvae pupated around August 25^th^. They pupated inside the millipede rather than exiting and pupating in the surrounding soil. It is unclear if this behaviour was due to laboratory conditions, or if it is also displayed in nature. Four males and four females emerged two weeks later, between 7 and 11 September 2017.

Descriptions and an identification key for males of S. (B.) cadyi and S. (B.) yorkii can be found in Giroux and Wheeler (2010). However, in order to help in the identification of these species, some digital photographs are provided here: the habitus of S. (B.) cadyi, male and female (Fig. 1A, B); the postabdomen of a S. (B.) cadyi male (Figs 1C, 2C, D) and female (Fig. 1E) as well as the one of a S. (B.) yorkii male (Fig. 3C, D). Male and female specimens of both species have tergite 5 with an orange-yellow posterior half or third (sometimes entirely yellow) and a row of strong setae forming a semi circle that spreads on the apical third (Fig. 1C–E). The male cerci and syntergosternite 7+8 are darker than the epandrium (Figs 1C, 2D, 3D). The window on male sternite 5 is almost even with the rest of the base (Figs 2C, 3C). The male distiphallus of both species as well as the external terminalia of female S. (B.) cadyi were digitally photographed (Figs 2A, 3A, 4A) and illustrations (Figs 2B, 3B, 4B) were added for a better understanding of their structural morphology.

## References

[B1] Aldrich JM (1916) *Sarcophaga* and allies in North America. The Thomas Say Foundation of the Entomological Society of America 1: 1–301. 10.5962/bhl.title.8573

[B2] Buenaventura E Pape T (2017) Phylogeny, evolution and male terminalia functionality of Sarcophaginae (Diptera: Sarcophagidae). Zoological Journal of the Linnean Society 183(4): 808–906. 10.1093/zoolinnean/zlx070

[B3] Buenaventura E Whitmore D Pape T (2017) Molecular phylogeny of the hyperdiverse genus *Sarcophaga* (Diptera: Sarcophagidae), and comparison between algorithms for identification of rogue taxa. Cladistics 33(2): 109–133. 10.1111/cla.12161 34710974

[B4] Coupland JB Barker GM (2004) Diptera as predators and parasitoids of terrestrial gastropods, with emphasis on Phoridae, Calliphoridae, Sarcophagidae, Muscidae and Fanniidae. In: Barker GM (Ed.) Natural Enemies of Terrestrial Molluscs.CAB International, Wallingford, 85–158. 10.1079/9780851993195.0085

[B5] Dahlem GA Naczi RFC (2006) Flesh flies (Diptera: Sarcophagidae) associated with North American pitcher plants (Sarraceniaceae), with description of three new species. Annals of the Entomological Society of America 99(2): 218–240. 10.1603/0013-8746(2006)099[0218:FFDSAW]2.0.CO;2

[B6] Giroux M Wheeler TA (2010) Systematics of *Bulbostyla*, a new subgenus of *Sarcophaga* Meigen, and change of status for *Robackina* Lopes (Diptera: Sarcophagidae). Zootaxa 2553(1): 35–59. 10.11646/zootaxa.2553.1.2

[B7] Hash JM Millar JG Heraty JM Harwood JF Brown BV (2017) Millipede defensive compounds are a double-edged sword: natural history of the millipede-parasitic genus *Myriophora* Brown (Diptera: Phoridae). Journal of Chemical Ecology 43(2): 198–206. 10.1007/s10886-016-0815-7 28078624

[B8] Mello-Patiu CA (2016) Family Sarcophagidae. Zootaxa 4122(1): 884–903. 10.11646/zootaxa.4122.1.75 27395323

[B9] Pape T (1990a) Two new species of *Sarcophaga* Meigen from Madeira and mainland Portugal (Diptera: Sarcophagidae). Tijdschrift voor Entomologie 133(1): 39–42. http://biostor.org/reference/49964

[B10] Pape T (1990b) Revisionary notes on American Sarcophaginae (Diptera: Sarcophagidae). Tijdschrift voor Entomologie 133(1): 43–74. https://archive.org/details/cbarchive_48786_revisionarynotesonamericansarc1990/page/n2

[B11] Pape T (1994) The world *Blaesoxipha* Loew, 1861 (Diptera: Sarcophagidae). Entomologica Scandinavica, Supplement 45: 1–247.

[B12] Pape T (1996) Catalogue of the Sarcophagidae of the world (Insecta: Diptera). Memoirs on Entomology 8: 1–557.

[B13] Ratnasingham S Hebert PDN (2007) The Barcode of Life Data System (http://www.barcodinglife.org). Molecular Ecology Notes 7(3): 355–364. 10.1111/j.1471-8286.2007.01678.x PMC1890991 18784790

